# Distal locked versus unlocked intramedullary nailing for stable intertrochanteric fractures, a systematic review and meta-analysis

**DOI:** 10.1186/s12891-020-03444-6

**Published:** 2020-07-13

**Authors:** Yan-Hui Li, Tiecheng Yu, Wenjing Shao, Yanwei Liu, Dong Zhu, Lei Tan

**Affiliations:** 1 Department of Cardiology and Echocardiography, the First Hospital of Jilin University, Changchun, 130021 China; 2 Department of Orthopedic Trauma, the First Hospital of Jilin University, No. 71 Xinmin Street, Changchun, 130021 Jilin China; 3 Department of Obstetrics and Gynecology, the First Hospital of Jilin University, Changchun, 130021 China

**Keywords:** Intramedullary nails, Intertrochanteric fracture, Locked intramedullary nailing, Meta-analysis, Outcomes, Unlocked intramedullary nailing

## Abstract

**Background:**

Intramedullary nails have become the main treatment for intertrochanteric fractures. However, a distal locking procedure during nailing gradually raised controversy. In this study, a systematic review and meta-analysis of clinical trials was performed to summarize existing evidence, aiming to determine the safety and efficacy of distal locking or unlocking in the nailing of stable intertrochanteric fractures.

**Methods:**

Appropriate articles were identified using the most common public databases, such as PubMed, Embase, the Cochrane Library, and Google Scholar from the inception of each database to April 2019, without restriction of language, publication date, and considering ongoing trials. Eligible studies were represented by randomized controlled trials or retrospective cohort studies, comparing distal locking and unlocking for the treatment of acute stable intertrochanteric fractures in adult patients. Information regarding methodological quality, patient demographics, and clinical outcomes were extracted independently by two reviewers. Subsequently, patients were divided into a locking and unlocking group.

**Results:**

This study included 9 articles, comprising a total of 1978 patients with a similar baseline. The results showed that the unlocking group had a shorter operation time, less intraoperative bleeding, lower transfusion rate, and less thigh pain after the treatment of femoral intertrochanteric fracture when compared with the distal locking group. No significant differences were observed in safety-related outcomes, including mortality, infection rate, cutting out, loss of reduction, backing out of lag screws, cephalic screw breakage, nail breakage, and peri-implant fractures between the two groups. In addition, efficacy-related outcomes including nonunion, delayed healing rates, and the Harris functional score were not significantly different between the two groups.

**Conclusions:**

Our pooled analysis demonstrated that distal unlocking of stable intertrochanteric fractures can shorten the operation time, reduce intraoperative bleeding, and reduce the blood transfusion rate. The use of locked or unlocked intramedullary nailing does not affect long-term outcomes regarding complications and function.

## Background

Intertrochanteric fracture of the femur is an extracapsular fracture occurring between the greater and lesser trochanters. Most patients are elderly individuals, suffering from other diseases and severe osteoporosis, thus, it is difficult to achieve satisfactory clinical results using the current available remedies. With the continuous progress of the aging of the human population, the incidence of intertrochanteric fracture increases annually [1]. Epidemiological survey data revealed a total of 1.66 million patients with hip fractures secondary to osteoporosis worldwide in 1990, which is expected to reach 6.26 million by 2050 [2].

The treatment of femoral intertrochanteric fractures has always been a challenge in orthopaedic surgery. Early surgical treatment can allow for an early start of functional exercises, thereby reducing complications caused by long-term bed rest, reducing disability and mortality, and improving the patients’ quality of life. At present, the intramedullary nail fixation system, including gamma nails (Stryker), InterTAN (Smith & Nephew), PFNA (Synthes), and TFN (Synthes) is more commonly used when compared to extramedullary implants. Advantages include small surgical trauma, short surgery time, and firm bone fixation. Furthermore, intramedullary nails may be beneficial in the treatment of unstable and subtrochanteric fractures [3]. Over the last 15 years, a striking increase in the use of intramedullary nails was observed, from 3 to 67% in Western countries [4, 5].

A distal locking screw of the intramedullary nail was designed to control proximal rotation and fracture shortening, and to prevent axial and rotational instability. Although distal locking is a routine procedure for intramedullary fixation in the treatment of intertrochanteric fractures, it has some disadvantages. Therefore, studies were performed to evaluate whether distal locking was necessary for intertrochanteric fractures [6–8]. Biomechanical studies revealed that distal locking of stable intertrochanteric fractures may not be required [6]. Rosenblum et al. found that the use of distal locking screw does not change femoral stress load for stable intertrochanteric fractures, and the tension of the proximal femoral bone does not change [6].

Various complications have been highlighted in the use of distal locking of intramedullary nails, including fascia lata irritation, additional operative time, intraoperative bleeding, radiation exposure, superficial femoral artery tear, implant loosening, and secondary femoral fractures [9, 10]. Thigh pain, erosion of the femoral cortex, and femoral fracture are consequences of the stress load at the distal screw [7]. Simmermacher et al. highlighted that an imprecise aiming device can weaken the femur and increase stress at the head of the locking screw [8]. Therefore, controversies still exist regarding the use of distal locking of intramedullary nails for stable intertrochanteric fractures.

To more comprehensively evaluate the therapeutic effect of the two methods, in this study, a meta-analysis was carried out based on all randomized controlled trials and retrospective cohort studies for the treatment of femoral intertrochanteric fractures, to provide medical evidence for a better clinical guide and performance.

## Methods

The work performed was in line with PRISMA (Preferred Reporting Items for Systematic Reviews and Meta-Analyses) and AMSTAR (Assessing the methodological quality of systematic reviews) guidelines.

### Search strategy

Relevant articles were identified through a computerized search in PubMed, Embase, the Cochrane Library, and Google Scholar. The keywords used were as follows: “intertrochanteric fracture” or “extracapsular hip fracture” or “peritrochanteric fracture” or “pertrochanteric fracture”, “nail”; and “locked” or “locking” or “unlocked” or “locking”. No restriction of language and publication date was applied, and ongoing trials were also considered.

### Inclusion and exclusion criteria

Inclusion criteria were as follows: (1) randomized controlled trials or retrospective cohort studies comparing locked intramedullary nailing (LIN) with unlocked intramedullary nailing (ULN) in the treatment of acute (treatment within 15 days from the trauma) intertrochanteric fractures (31-A1, and A2, 2) patients older than 18 years; (3) studies reporting at least one of the main outcomes, such as operation length, intraoperative blood loss, transfusion rate, length of hospital stay, peri-implant fracture rate, reoperation rate, mortality rate, Harris hip scores (1 year), and complications (infection, cutting out, loss of reduction, backing out of lag screws, cephalic screw breakage, nail breakage, nonunion and delayed healing rates, femoral head necrosis).

Exclusion criteria were as follows: open fracture, bilateral fractures, pathological fracture; duplicated or overlapping data; dissimilar demographic background of patients and preoperative conditions; cadaver or model studies; unreported data.

### Data extraction and quality assessment

Independent literature screening, data extraction and quality evaluation were performed by two authors (YL & LT), and were cross-checked. A preliminary screening of the title and abstract was conducted to remove studies that were significantly beyond the scope of the study. In case of uncertainty based on the title or abstract, the full text of each study was obtained for further evaluation. Data from studies that met the inclusion criteria were extracted. The two authors mentioned above independently completed the data extraction as required by this review. A third author (DS) independently assessed all studies for eligibility and inclusion. Discussion among the authors was used to solve any inconsistencies.

### Risk of bias assessment in included studies

The Cochrane handbook was used as the quality evaluation standard for randomized controlled trials. The Newcastle-Ottawa scale (NOS) was used to assess non-randomized controlled trial studies [11]. A quality score ≥ 7 on the nine-point of the NOS was considered of relatively high quality for cohort studies. Subgroup analysis was mainly carried out according to the research design scheme. Subgroup analysis of different fracture types was not performed, because a limited number of articles had data describing different fracture types.

### Data synthesis

The relative risk or odd ratio was calculated for results that were discrete variables, and the mean difference was used when results were continuous variables; 95% CI was determined for all effect sizes. Heterogeneity test was based on Cochran’s Q statistic χ^2^ test and I^2^ test. The χ^2^ test used *P* < 0.05 as the test standard, and I^2^ used < 50% as the test standard. The fixed effect model was used to analyse the results when no statistical heterogeneity was present, and the random effect model was used to analyse results when a statistical heterogeneity was present.

In addition, to confirm the reliability of the results of the meta-analysis, each of the studies included was excluded one time in turn, the remaining studies were combined and sensitivity analysis was carried out. When changes occurred, further analysis was performed to identify the cause of the heterogeneity, and therefore, results giving stability and strength were discovered. If the heterogeneity was too large to analyse, a descriptive analysis was performed. Funnel maps were drawn to test the publication bias of the included articles.

Review Manager 5.3 software (Cochrane Collaboration, London, UK) was used for the meta-analysis performed in this study. *P* < 0.05 was considered statistically significant.

## Results

The work performed was in line with PRISMA (Preferred Reporting Items for Systematic Reviews and Meta-Analyses) and AMSTAR (Assessing the methodological quality of systematic reviews) guidelines.

### Study characteristics and quality assessment

**Fig. 1:**
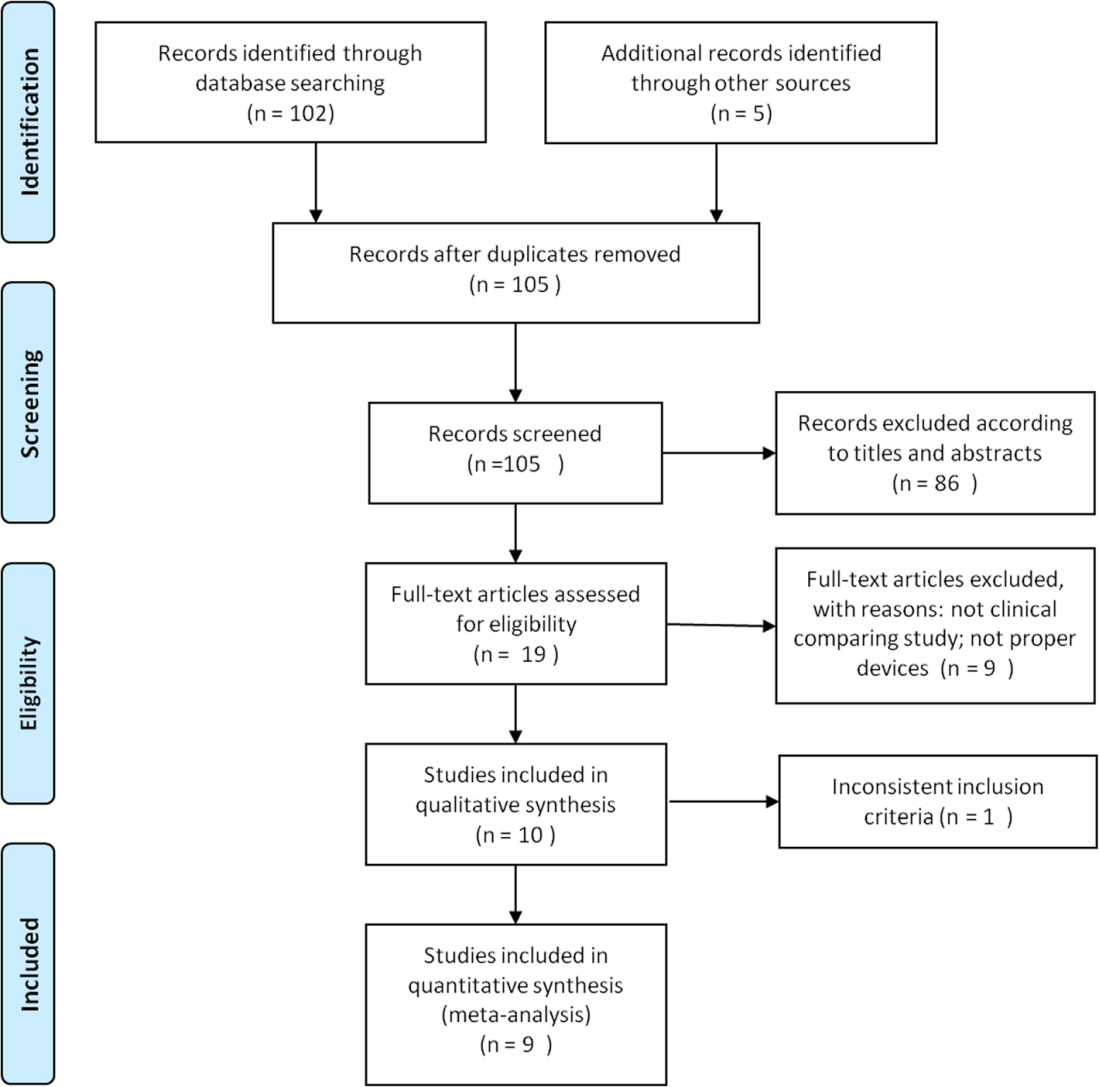
Study flow diagram

Literature search and screening results led to a total of 105 articles. Eighty-six articles were excluded after reading the abstracts. Another 10 unrelated biomechanical studies were excluded after further reading. Finally, 9 studies (4 randomized controlled trials [12–15] and 5 observation studies [16–20]) met our inclusion and exclusion criteria, including 7 in English [12–14, 16–19], 1 in Spanish [15], and 1 in Korean [20] (Fig. 1).

### Quality assessment

**Fig. 2:**
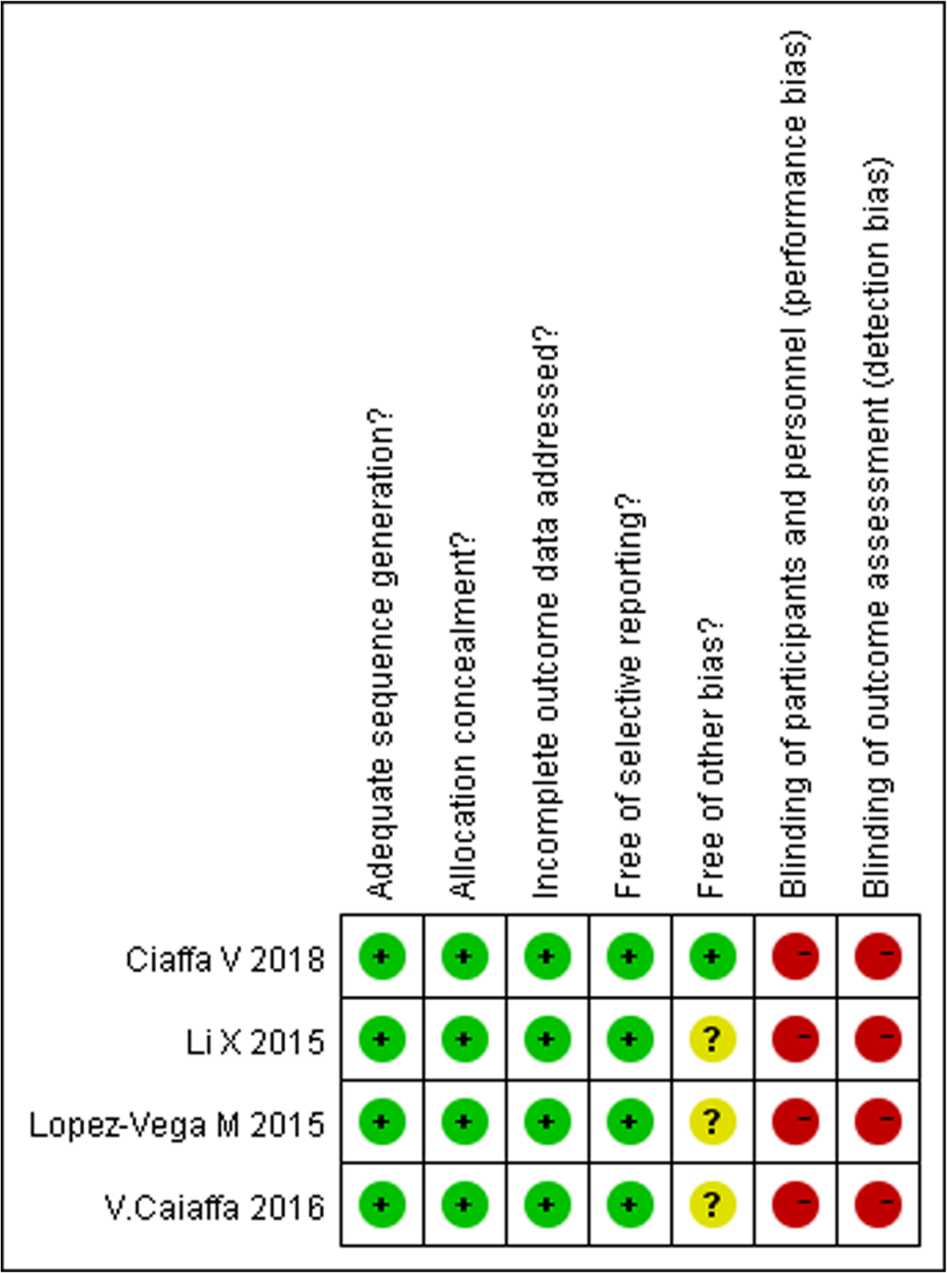
Summary of the risk of bias of each included randomized controlled trial

Included randomized controlled trials [12–15] reported an adequate generation of the allocation sequence and concealment, however surgeons blinded to the surgical intervention were not present in the design study. In all these studies, the evaluation process of the outcome was also not blinded. Loss to follow-up due to factors, such as death and inability to move was found in all four studies. However, missing outcome data balanced in numbers across intervention groups, with similar reasons for missing data across groups. No studies reported grants in support of their research (Fig. 2). The 5 cohort studies [16–20] were considered of relatively high quality because of a score ≥ 7 according to the NOS scale criteria (Table 1).

**Table 1:** Characteristics of the included studies

Study	Year	Nation	Study design	Diagnosis characteristics (AO/OTA)	Implant	Gender (M/F)		Mean age (years)		Type of fracture (A1/A2/total, n)		Follow-up (months)
						Locking	Locking	Unlocking	Unlocking	Locking	Unlocking	
Ciaffa V	2018	Italy	RCT	31-A1+ A2	Citieffe nail	89/50	77.1 ± 2.3	75.6 ± 3.5	49/24	46/93/139	25/48/73	12
Lanzetti RM	2018	Italy	PCS	31-A1+ A2	SM Supernail	11/57	84.5 ± 8.76	85.48 ± 7.84	13/62	38/30/68	35/40/75	14.1 (12–18).
Li X	2015	China	RCT	31-A1 + A2	PFNA-II	11/24	78.1 ± 6.9	78.3 ± 7.0	10/25	7/28/35	11/24/35	12
Lopez-Vega M	2015	Spain	RCT	31-A1 + A2	Gamma 3 nail	17/73	84,59 ± 9,11	83,68 ± 6,90	19/68	34/56/90	46/41/87	12
Skala-Rosenbaum	2010	Czech Republic	PCS	31A1 + A2	PFH	10/34	79.6	79.8	17/57	14/30/44	38/36/74	12
Skala-Rosenbaum J	2016	Czech Republic	PCS	31A1 + A2	PFN	N/A	N/A	81.7	82.8	NA/NA/595	NA/NA /254	12
V.Caiaffa	2016	Italy	RCT	31-A1 + A2	Citieffe nail	41/89	78.4 ± 7.1	77.9 ± 7.2	52/84	37/93/130	48/88/136	12
Vopat BG	2014	USA	RCS	31-A1.1,+.2, +.3	long Gamma3	NA	NA	NA	NA	56/0/56	51/0/56	12
Yun, Ho Hyun	2015	Korea	RCS	31-A1.1 + .2	Gamma3	11/7	75.1 ± 11.7	75.1 ± 11.7	21/8	18/0/18	29/0/29	17.8 ± 10.6

*RCT* randomized controlled trial, *PCS* prospective cohort study, *RCS* retrospective cohort study, *M/F* male/female, *NA* notavailable

### Demographic characteristics

In total, 1978 patients were included in this study, and divided into 1169 LIN patients and 809 ULN patients. The average follow-up was at least 1 year. Demographic characteristics are summarized in Table 2.

**Table 2:** Quality Assessment of Included Cohort Studies Using the Newcastle-Ottawa Scale

**Author**	**Selection**				**Comparability**		**Outcome**			
	**Representativeness of Exposed Cohort**	**Selection of Non-Exposed Cohort**	**Ascertainment of Exposure**	**Demonstration That Outcome of Interest Was Not Present at Start of Study**	**Adjust for fracture type**	**Adjust for other fracture risk factors**	**Assessment of outcome**	**Follow-up > 1 year**	**Loss to follow-up rate**	**Total Quality Score**
Lanzetti RM. 2018 [5]	1	1	1	1	1	0	1	1	1	8
Skala-Rosenbaum, 2010 [6]	1	1	1	1	1	0	1	1	1	8
Skala-Rosenbaum,2016 [7]	1	1	1	1	1	0	1	0	1	7
Vopat BG, 2014 [8]	1	1	1	1	0	0	1	1	1	7
Yun, Ho Hyun, 2015 [9]	1	1	1	1	1	0	0	1	1	7

The quality of included studies was assessed by the Newcastle Ottawa scale. A study can be awarded a maximum of one star for each numbered item within the Selection and Outcome categories and a maximum of two stars for Comparability

Selection: 1) Representativeness of exposed cohort: 1, study population truly or somewhat representative of a community/ population based study; 0, study population was sampled from a special population, that is, population from a company, hospital patients, data from the health insurance company or health examination organization, nurses

2) Selection of non-exposed cohort: 1, drawn from the same community as the exposed cohort

3) Ascertainment of exposure: 1, Validation of macrolides use with secure medical record; 0, no specific macrolides use validation method

4) Demonstration that outcome was not present at start of study: 1, exclusion of participants with a history of severe ventricular arrhythmia or sudden cardiac arrest at the beginning of the study

Comparability: 1) 1, whether a study adjusted for fracture type deliberately; 1, whether a study adjusted for other risk factors

Outcome: 1) Assessment of outcome: 1, events were confirmed by medical records or record linkage; 0, self-reported

2) Was follow-up long enough for outcomes to occur: 1, duration of follow-up > = 1 year; 0, if duration of follow-up < 1 year

3) Loss to follow-up rate: 1, complete follow-up or loss to follow up rate < =20%; 0, follow-up rate < 80% or no description of those lost

### Effects of interventions

#### Surgical parameters

**Fig. 3:**
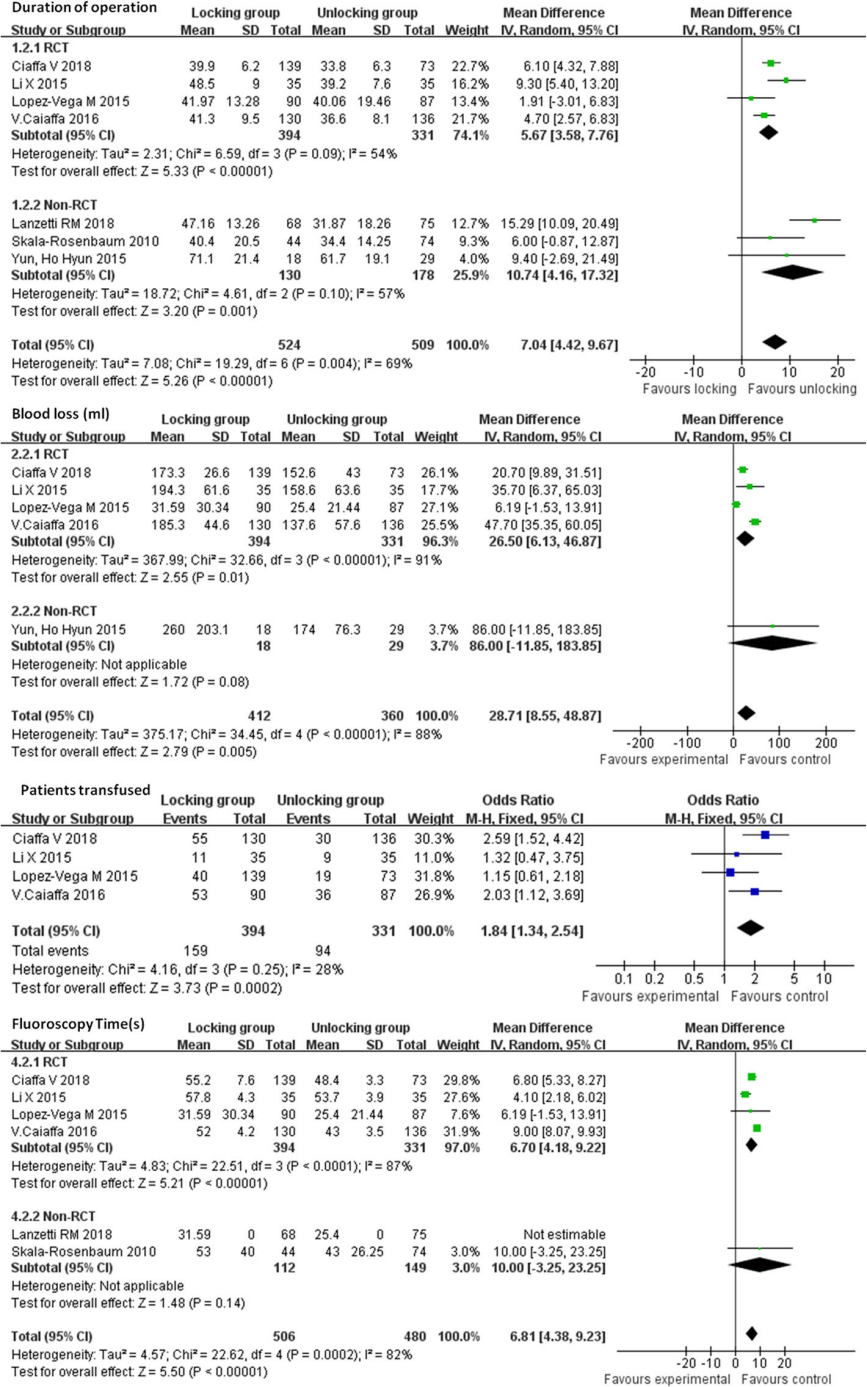
Comparison of the duration of operation, blood loss, patients transfused, and fluoroscopy time between the locking group and unlocking group. SD = standard deviation, IV = inverse variance, CI = confidence interval, and df = degrees of freedom

Duration of the operation, intraoperative blood loss, transfusion rate, and fluoroscopy time were recorded. Seven studies [12–17, 20] provided data regarding the duration of the operation. The data showed a significant difference in the duration of the operation between the two groups, and the average operation time when distal unlocking was performed was shorter when compared to that needed for performing distal locking (mean difference: 7.04, 95% CI: 4.42–9.67, *P* < 0.00001) (Fig. 3). Five studies [12–15, 20] provided data regarding intraoperative blood loss. The data showed that average intraoperative blood loss when distal unlocking was performed was less when compared to that after during distal locking (mean difference: 28.71, 95% CI: 8.55–48.87, *P* = 0.005) (Fig. 3). Six studies [12–17] provided data regarding intraoperative fluoroscopy time. The data showed that the average intraoperative fluoroscopy time during distal unlocking was less when compared to that during distal locking (mean difference: 9, 95% CI: 8.07–9.93, *P* < 0.00001) (Fig. 3). Heterogeneity existed among the studies. The random effect model was used for the meta-analysis in this study. In addition, each of the studies used was excluded one time in turn, and the remaining studies were combined for analysis. The heterogeneity remained unchanged, and the results of sensitivity analysis were reliable. Subgroup analysis based on the design type confirmed the above-mentioned results.

Six articles [12–15] included 705 fractures provided data regarding blood transfusion. No heterogeneity existed among the studies (*P* = 0.25; I^2^ = 28%). The fixed effect model was used for meta-analysis. The results regarding the rate of blood transfusion revealed significantly less blood transfusion in the distal unlocking group when compared to the locking group (Odd ratio: 1.84, 95% CI: 1.34–2.54, *P* = 0.0002) (Fig. 3).

#### Complications

All nine studies [12–20] provided data regarding complications. Statistically significant differences were not observed between distal locking and distal unlocking in total complications and subgroups, such as hematoma, deep vein thrombosis, avascular necrosis of the femoral head, and infection. Outcomes associated with implant failure and stability including cutting out, loss of reduction, backing out of lag screws, cephalic screw breakage, and nail breakage. Healing-related outcomes included nonunion and delayed healing rates. In addition, no significant differences were observed regarding the above-mentioned outcomes between the two groups. The pooled results are shown in Table 3. Outcomes including healing time and reoperation rate were not involved in the final result because only this was only reported in one document.

**Table 3:** Postoperative comparison of LIN and ULN

**Studies**	**No. of studies**	**Patients (N)**	**Distal locking (events/total)**	**Distal unlocking (events/total)**	**Heterogeneity (I**^**2**^, ***P***)	**Result OR /MD, 95%CI,*P***
**Total complications**						
RCT subgroup	2	261	73/258	50/190	I^2^ = 0%, *P* = 0.90	1.40 [0.90, 2.19] *P* = 0.14
Non-RCT subgroup	2	448	20/112	21/149	I^2^ = 0%, *P* = 0.34	1.28 [0.65, 2.51] *P* = 0.06
Overall	4	709	93/370	71/339	I^2^ = 0%, *P* = 0.88	1.36 [0.94, 1.98] *P* = 0.10
**Haematoma**	3	537	11/298	8/239	I^2^ = 0%, *P* = 0.78	1.13 [0.44, 2.90] *P* = 0.80
**Deep Vein Thrombosis**	3	537	3/298	2/239	I^2^ = 0%, *P* = 0.98	1.18 [0.22, 6.26] *P* = 0.85
**Cutting out**						
RCT subgroup	4	714	6/388	3/326	I^2^ = 0%, *P* = 0.96	1.51 [0.37, 6.25] *P* = 0.57
Non-RCT subgroup	4	415	2/186	1/229	I^2^ = 41%, *P* = 0.90	1.52 [0.26, 8.99] *P* = 0.64
Overall	8	1129	8/574	4/555	I^2^ = 0%, *P* = 0.64	1.52 [0.50, 4.60] *P* = 0.46
**Loss of reductio**n						
RCT subgroup	3	655	3/421	5/399	I^2^ = 43%, *P* = 0.19	0.66 [0.08, 5.76] *P* = 0.71
Non-RCT subgroup	2	165	3/359	5/296	N/A	N/A
Overall	5	820	0/62	0/103	I^2^ = 43%, *P* = 0.19	0.66 [0.08, 5.76] *P* = 0.71
**Peri-implant fractures**						
RCT subgroup	4	714	6/388	8/326	I^2^ = 60%, *P* = 0.11	0.50 [0.06, 4.23] *P* = 0.52
Non-RCT subgroup	4	1157	5/725	18/432	I^2^ = 78%, *P* = 0.004	0.73 [0.05, 11.03] *P* = 0.82
Overall	8	1871	11/1113	26/758	I^2^ = 69%, *P* = 0.006	0.59 [0.11, 3.18] *P* = 0.54
**Wound infection**	4	714	12/388	13/326	I^2^ = 0%, *P* = 0.80	0.76 [0.34, 1.67] *P* = 0.49
**Deep infection**	4	714	3/388	0/326	N/A	7.00 [0.36, 137.53] *P* = 0.20
**Delayed union**						
RCT subgroup	4	714	5/388	3/326	I^2^ = 41%, *P* = 0.18	1.53 [0.43, 5.43] *P* = 0.51
Non-RCT subgroup	3	308	0/130	1/178	N/A	0.51 [0.02, 13.29] *P* = 0.69
Overall	7	1022	5/518	4/504	I^2^ = 30%, *P* = 0.19	1.30 [0.41, 4.13] *P* = 0.65
**Non-union**	3	272	0/298	1/239	N/A	0.35 [0.01, 8.57] *P* = 0.52
**Avascular necrosis of the femoral head**						
RCT subgroup	3	655	1/359	2/296	I^2^ = 0%, *P* = 0.82	0.41 [0.05, 3.30] *P* = 0.40
Non-RCT subgroup	3	368	0/168	2/200	I^2^ = 0%, *P* = 0.79	0.40 [0.04, 3.93] *P* = 0.43
Overall	6	1023	1/527	4/496	I^2^ = 0%, *P* = 0.99	0.41 [0.09, 1.90] *P* = 0.25
**Thigh pain**						
RCT subgroup	3	502	49/249	16/253	I^2^ = 63%, *P* = 0.07	2.90 [0.97, 8.68] *P* = 0.06
Non-RCT subgroup	3	272	7/118	6/154	I^2^ = 20%, *P* = 0.29	1.62 [0.37, 7.04] *P* = 0.52
Overall	6	774	55/367	22/407	I^2^ = 45%, *P* = 0.10	2.45 [1.05, 5.73] *P* = 0.04

**Fig. 4:**
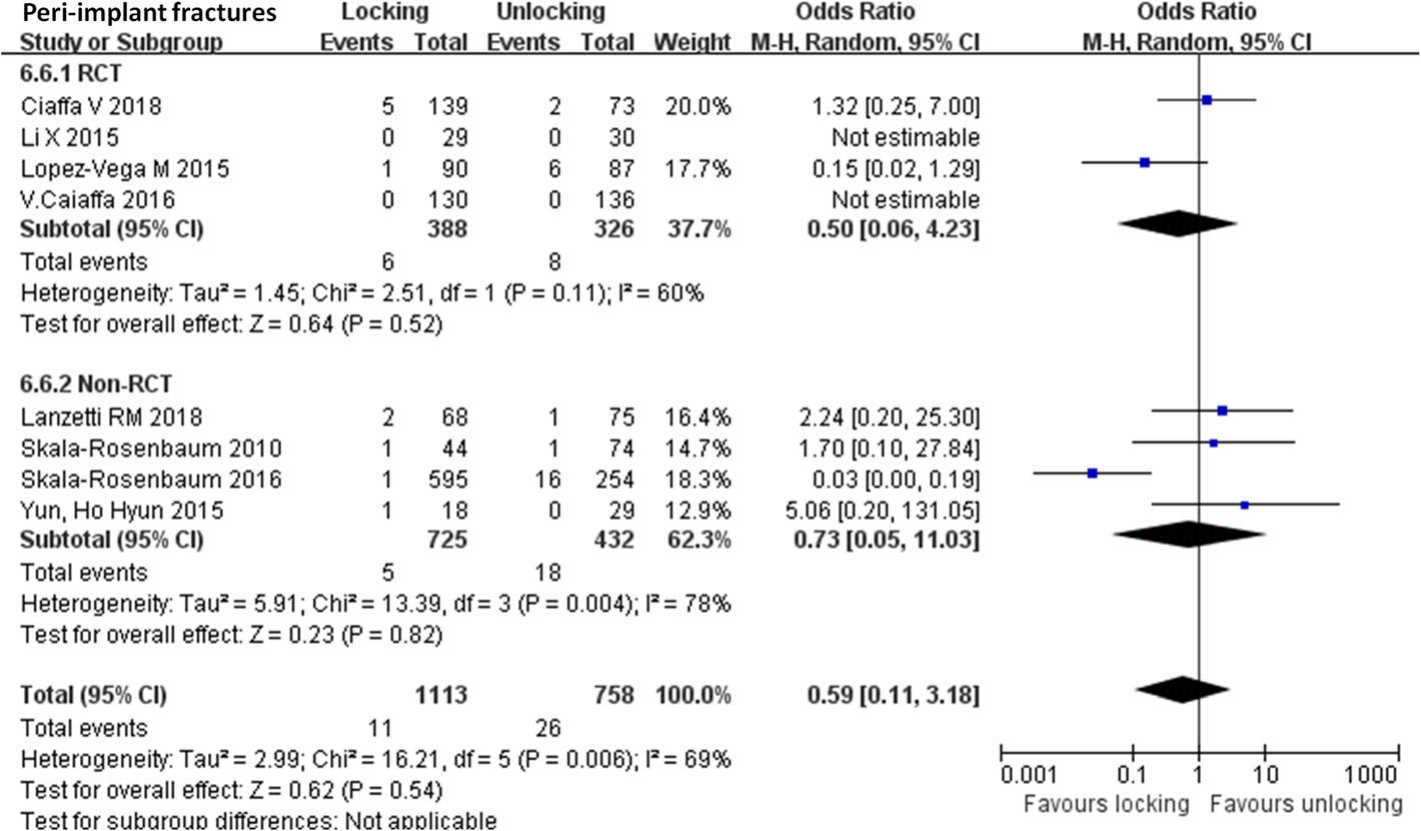
Comparison of peri-implant fractures between the locking group and the unlocking group. SD = standard deviation, IV = inverse variance, CI = confidence interval, and df = degrees of freedom

Eight articles [12–18, 20] provided results regarding peri-implant fractures. Heterogeneity existed among the studies (*P* = 0.006; I^2^ = 69%). The random effect model was used for meta-analysis. A total of 1871 fractures were included, divided into 1113 patients with distal locking and 758 without distal locking. The results did not show significant differences in peri-implant fractures between the two groups (Odd ratio: 0.72, 95% CI: 0.14–3.82, *P* = 0.070) (Fig. 4). In addition, each of the studies used was excluded one time in turn, and the remaining studies were combined for analysis. The heterogeneity decreased significantly when the study of Skala-Rosenbaum [18] was excluded (*P* = 0.82; I^2^ = 0%), and the results remained not statistically different in peri-implant fractures between the two groups (Odd ratio: 1.43, 95% CI: 0.51–4.06, *P* = 0.050). Subgroup analysis based on the design type confirmed the above-mentioned results.

#### Mortality

**Fig. 5:**
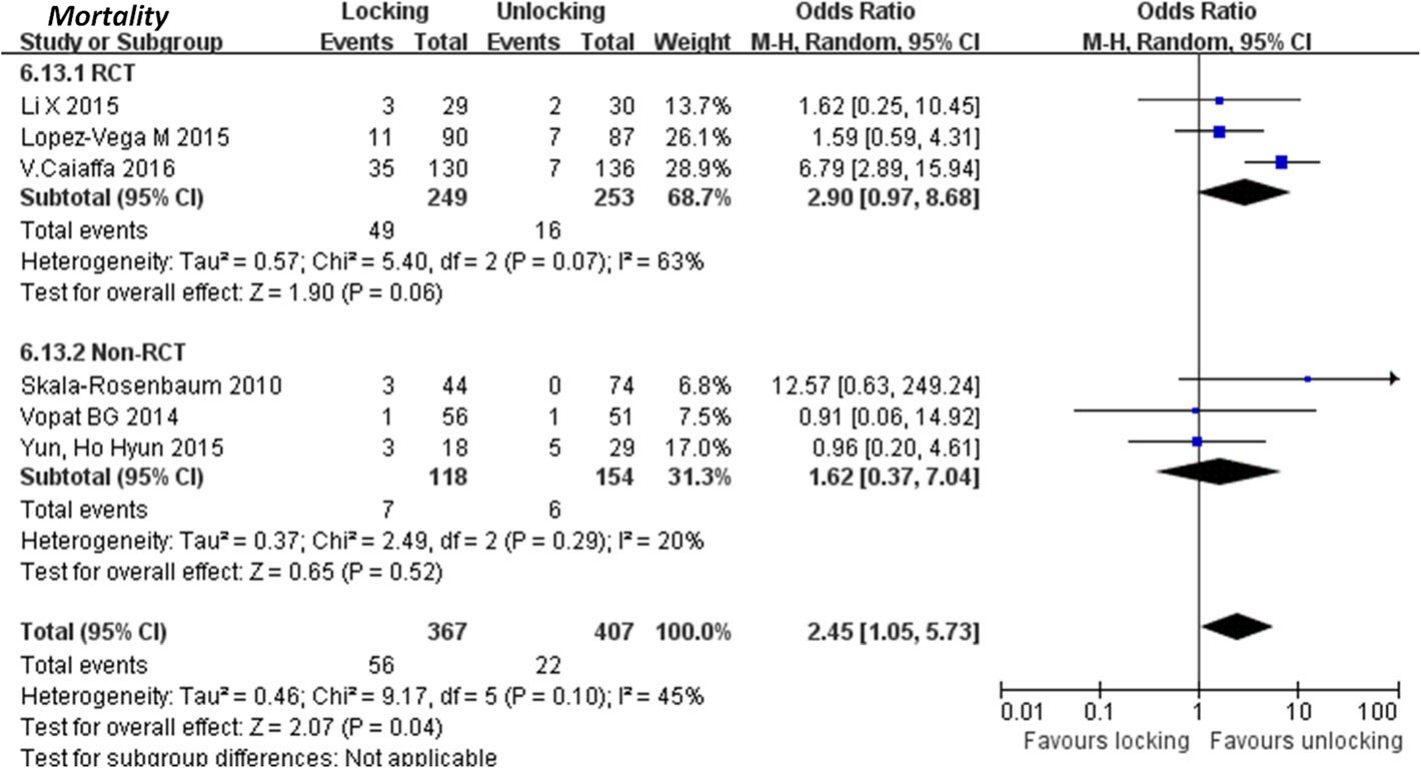
Comparison of mortality between the locking group and the unlocking group. SD = standard deviation, IV = inverse variance, CI = confidence interval, and df = degrees of freedom

Four articles [12–15] provided results regarding mortality. No heterogeneity existed among the studies (*P* = 0.41; I^2^ = 0%). The fixed effect model was used for meta-analysis. A total of 714 fractures were included and divided into 388 patients with distal locking and 326 without distal locking. The results did not show significant differences in mortality between the two groups (Risk ratio: 1.30, 95% CI: 0.94–1.82, *P* = 0.12) (Fig. 5).

#### Function

Four articles provided results regarding the function 1 year post-operation [12–15]. The results did not show differences in Harris hip score, rates of normal walking ability, walking with a walking aid, and the use of a wheelchair between the two groups. The pooled results are shown in Table 4.

**Table 4:** Function comparison of LIN and ULN

**Studies**	**No. of studies**	**Patients (N)**	**Distal locking (events/total)**	**Distal unlocking (events/total)**	**Heterogeneity (I**^**2**^, ***P*)**	**Result OR/RR /MD, 95%CI,*P***
**Harris Hip Score**	3	537	N/A	N/A	I^2^ = 35%, *P* = 0.22	0.14 [−1.33, 1.62] *P* = 0.85
**Normal walking ability**	4	714	242/388	224/326	I^2^ = 82%, *P* = 0.0009	0.89 [0.68, 1.17] *P* = 0.41
**Walk with a walking aid**	3	537	61/298	50/239	I^2^ = 0%, *P* = 0.91	0.91 [0.65, 1.28] *P* = 0.59
**Use of wheelchair**	3	537	22/298	18/239	I^2^ = 0%, *P* = 0.83	1.07 [0.55, 2.07] *P* = 0.84
**Mortality**	4	714	71/388	49/326	I^2^ = 0%, *P* = 0.41	1.30 [0.94, 1.82] *P* = 0.12

## Discussion

Intramedullary nails have become the mainstream treatment for intertrochanteric fractures due to their advantages of being minimally invasive and biomechanics features. However, with the widespread use of intramedullary nails, the distal locking procedure as a routine operation gradually raised controversy. Complications emerged associated with distal locking, including fascia lata irritation, secondary femoral fractures [5, 9, 10], thigh pain, and erosion of the femoral cortex, femoral cortical hypertrophy and superficial femoral artery [8]. Due to this emerging problem, in some studies the utility of using distal interlocking screws in their biomechanical studies was investigated, demonstrating that distal locking was unnecessary in stable and in some unstable intertrochanteric fractures [2, 6, 21–25]. In many studies, the outcomes of distal locking and unlocking in the intramedullary nailing for the treatment of intertrochanteric fractures was compared, however, clear inconsistency in treatment effects were described in these studies. Thus, the optimal method to deal with distal locking during nailing of intertrochanteric fractures remains controversial [12–20]. Therefore, the purpose of this meta-analysis and systematic review was to summarize existing evidence to determine the safety and efficacy of distal locking and unlocking in the nailing of intertrochanteric fractures. To our knowledge, no similar meta-analysis has been performed.

### Summary of evidence

This study considered 9 articles that included a total of 1978 patients with a similar baseline. The results showed that the distal unlocking group had a shorter operation time, less intraoperative bleeding, transfusion rate, and thigh pain in the treatment of femoral intertrochanteric fracture when compared with the distal locking group. No significant differences in safety-related outcomes, including mortality, infection rate, cutting out, loss of reduction, backing out of lag screws, cephalic screw breakage, nail breakage, and peri-implant fractures was found. In addition, efficacy-related outcomes, including nonunion, delayed healing rates, and the Harris functional score were not significantly different. According to the GRADE tool, most of these outcomes were graded as low-moderate.

Consistent with the foregoing expected results, the operative time, blood loss, rate for blood transfusion, and radiation exposure in ULN were significantly reduced when compared with LIN. The operating time was shorter and radiation exposure was les because of less surgical procedures. The reason for the low amount of blood loss was mainly due to smaller trauma and a shorter operation time. The short operation time and the small amount of intraoperative blood loss minimize the anaesthetic effect on respiration and blood circulation, and represent an advantage for the recovery of elderly patients after surgery. Reduced transfusion implies a reduced risk of disease transmission, transfusion reactions, and immunomodulation, and it reduces the costs of the transfusions [26]. Less radiation exposure can reduce harm and increase the protection for both patients and surgeons. A statistically significant difference of heterogeneity existed due to different hospitals that calculated the operating time and blood loss, different internal fixation, and the inconsistency of surgeon proficiency.

The overall results regarding thigh pain were significant, suggesting that the use of distal screw might be harmful. This is consistent literature reports [12, 15, 16, 18]. However, when subgroup analysis was performed, differences were not significant, suggesting that the reliability of these findings was not strong.

Two approaches exist to lock distal nails: static locking and dynamic locking. Most included studies did not distinguish between the two approaches. In the study by Ciaffa et al., their prospect comparative analysis was expanded, including static locking vs dynamic locking vs no locking. No significant differences were observed across the three groups regarding major radiological performancce of fracture union, malunion, as well as regarding HHS, SF-12 and Barthel index results after 1-year follow-up [13].

The occurrence of cut-out and nonunion after cephalomedullary nailing of stable pertrochanteric fractures appeared to be correlated to the presence of cortical impingement [27]. Therefore, a fake unlocked femoral nail with cortical impingement should be avoided in stable intertrochanteric femur fractures.

### Disagreements with other studies

Rates of peri-implant fractures were similar in both LIN and ULN groups and therefore not statistically significant. However, the data were heterogeneous (*P* = 0.010; I^2^ = 67%), mainly due to the Skala-Rosenbaum et al. study [18]. After removal of this article, although the conclusion remains unchanged, the heterogeneity significantly decreased (*P* = 0.82; I^2^ = 0%). In the present study, after analysing a group of 849 pertrochanteric fractures managed with short nails, we found that patients without distal locking had an 85.7% higher risk of peri-implant fractures. This finding is different from the results of all clinical and biomechanical studies considered in our work. Methodologically speaking, the reason of this difference may be due to the absence of random and blind methods in the study by Skala-Rosenbaum et al., and in addition, the baseline and weight-bearing time between the two groups were not introduced. Clinically speaking, the difference might theoretically be related to the fracture type or the inappropriate selection of patients for unlocked nailing, whose fracture should be a stable fracture for this choice of using unlocked nailing, because the dorsomedial fragment or the existence of recessive fracture is often difficult to detect by ordinary X-ray evaluation [18, 28]. In addition, peri-implant fractures are associated with instability. The stability is not only related to internal fixation, but also to surgical reduction. Poor surgical reduction can also lead to instability [29]. The study by Skala-Rosenbaum et al. did not mention the effect of post-operative reduction. Furthermore, their study did not give a detailed description of the tip apex distance and the position of the head nail, which are closely related to the stability.

### Strengths and limitations

This meta-analysis has some limitations. 1. The nine articles included 1978 cases of intertrochanteric fractures of the femur. Five of them were observational studies. Some defects were present in the research design, and the performance of the statistical tests may be insufficient. 2. The nine articles involved did not describe each measurement and outcome in detail, and the validity of the statistical tests may be insufficient. We tried to contact the authors of the included studies for more information, however, we did not receive any response regarding the possibility to check the data of their study. Therefore, subgroup analysis of some aspects, including fracture types, intramedullary nails, and complications to rule out possible confounding factors was not performed, thereby affecting the effectiveness of our study. 3. The inconsistency about nails length, number or type of cephalic screws and angle between cephalic screws are unclear aspects that make our results questionable.

Further research should be performed considering larger, multicenter, randomized controlled studies that take into account the need for large clinical trials with a valid, type-specific fracture and uniform method for the measurement and definition of the outcome. We recommend that CT should be performed to identify the type of fracture as stable before deciding to use distal locking nails. Patients with wide diameters of the medullary cavity, comminution of the lateral wall of the greater trochanter, and large posteromedial fragment extending distally below the lesser trochanter should not be considered for unlocked intramedullary nailing. Current studies mainly focus on short nails, but relatively few on long nails. Therefore, this study of long intramedullary nailing for intertrochanteric treatment should be properly enriched with other studies performed as recommended above.

## Conclusions

In our systematic review, we showed that the distal unlocking of the intertrochanteric fractures can shorten the operation time, reduce intraoperative bleeding, and reduce the blood transfusion rate. The choice between the locking or unlocking procedure does not affect long-term outcomes regarding complications and function.

## Data Availability

The dataset supporting the conclusions of this article is included within the article.

## Abbreviations

PFNA: Proximal Femoral Nail Antirotation
TFN: Trochanteric Fixation Nail
PRISMA: Preferred Reporting Items for Systematic Reviews and Meta-Analyses
AMSTAR: Assessing the methodological quality of systematic reviews
LIN: Locked Intramedullary Nailing
ULN: Unlocked Intramedullary Nailing
